# Prevalence of Micronutrient Deficiencies in Patients Hospitalized with COVID-19: An Observational Cohort Study

**DOI:** 10.3390/nu14091862

**Published:** 2022-04-29

**Authors:** Manyola Voelkle, Claudia Gregoriano, Peter Neyer, Daniel Koch, Alexander Kutz, Luca Bernasconi, Anna Conen, Beat Mueller, Philipp Schuetz

**Affiliations:** 1 Medical University Department of Medicine, Kantonsspital Aarau, 5001 Aarau, Switzerland; manyola.voelkle@ksa.ch (M.V.); claudia.gregoriano@ksa.ch (C.G.); daniel.koch@ksa.ch (D.K.); alexander.kutz@ksa.ch (A.K.); beat.mueller@ksa.ch (B.M.); 2 Faculty of Medicine, University of Basel, 4056 Basel, Switzerland; anna.conen@ksa.ch; 3 Institute of Laboratory Medicine, Kantonsspital Aarau, 5001 Aarau, Switzerland; peter.neyer@ksa.ch (P.N.); luca.bernasconi@ksa.ch (L.B.); 4 Department of Infectious Diseases and Infection Prevention, Kantonsspital Aarau, 5001 Aarau, Switzerland

**Keywords:** COVID-19, micronutrients, deficiency, SARS-CoV-2, hospital outcomes

## Abstract

Background: A higher risk for severe clinical courses of coronavirus disease 2019 (COVID-19) has been linked to deficiencies of several micronutrients. We therefore studied the prevalence of deficiencies of eight different micronutrients in a cohort of hospitalized COVID-19-patients. Methods: We measured admission serum/plasma levels of vitamins A, B12, D, and E, as well as folic acid, zinc, selenium, and copper in 57 consecutively admitted adult patients with confirmed COVID-19 and analyzed prevalence of micronutrient deficiencies and correlations among micronutrient levels. Further, we studied associations of micronutrient levels with severe disease progression, a composite endpoint consisting of in-hospital mortality and/or need for intensive care unit (ICU) treatment with logistic regression. Results: Median age was 67.0 years (IQR 60.0, 74.2) and 60% (*n* = 34) were male. Overall, 79% (*n* = 45) of patients had at least one deficient micronutrient level and 33% (*n* = 19) had ≥3 deficiencies. Most prevalent deficiencies were found for selenium, vitamin D, vitamin A, and zinc (51%, 40%, 39%, and 39%, respectively). We found several correlations among micronutrients with correlation coefficients ranging from r = 0.27 to r = 0.42. The strongest associations with lower risk for severe COVID-19 disease progression (adjusted odds ratios) were found for higher levels of vitamin A (0.18, 95% CI 0.05–0.69, *p* = 0.01), zinc (0.73, 95% CI 0.55–0.98, *p* = 0.03), and folic acid (0.88, 95% CI 0.78–0.98, *p* = 0.02). Conclusions: We found a high prevalence of micronutrient deficiencies in mostly older patients hospitalized for COVID-19, particularly regarding selenium, vitamin D, vitamin A, and zinc. Several deficiencies were associated with a higher risk for more severe COVID-19 courses. Whether supplementation of micronutrients is useful for prevention of severe clinical courses or treatment of COVID-19 warrants further research.

## 1. Introduction

Over the last two years, severe acute respiratory syndrome coronavirus 2 (SARS-CoV-2) led to a global pandemic with high morbidity and mortality, causing over 426 million infections and almost 6 million deaths up to February 2022 [1]. Clinical courses range from asymptomatic infection to severe disease with need for intensive care unit (ICU) stay and death [2]. Different risk factors for severe COVID-19 have been described and include older age, frailty, and higher burden of comorbidities [3,4]. However, because older and frail patients are often malnourished and have a higher likelihood for low levels in different specific micronutrients [5,6], micronutrient deficiencies could additionally contribute to more severe courses in patients infected with SARS-CoV-2. From a preclinical perspective, the importance of various micronutrients for a functioning immune system has been well documented [7,8,9]. For example, it has been shown that vitamin A plays an important role in maintaining mucosal integrity [10], while zinc plays an essential role in protecting against reactive oxygen and nitrogen species [11,12]. Furthermore, vitamin D increases the excretion of antimicrobial peptides in epithelial lining cells in the respiratory tract [13] and is involved in the modulation of pro- and anti-inflammatory cytokine production [14]. Consequently, deficiencies in micronutrients may lead to higher susceptibility for infections.

With respect to COVID-19, deficiencies in different micronutrients, including vitamin D [15,16], zinc [17,18], and selenium [19,20], have been discussed as risk factors for a more severe disease course, with need for ICU admission and mechanical ventilation, or higher incidence of death. Based on these observations, there has been a call for more wide-spread supplementation of the above-mentioned micronutrients during the COVID-19 pandemic to prevent and improve courses of infected patients [7,21]. This call is particularly timely, since a high prevalence of deficiencies has been reported from different countries, including Switzerland [22,23]. Still, until now, there is insufficient research concerning the association of micronutrients levels with clinical courses of COVID-19.

Herein, we analyzed different micronutrient levels in a COVID-19 cohort and described the distribution of deficiencies, as well as the correlation among levels of different micronutrients. Further, the association between deficiencies in micronutrients and severe progression of COVID-19 disease was investigated.

## 2. Subjects and Methods

### 2.1. Patient Population

This prospective observational study involved adult patients (≥18 years) hospitalized with a confirmed SARS-CoV-2 infection between 17 March 2020 and 30 April 2020 at the Cantonal Hospital Aarau (Switzerland), a tertiary care hospital. Baseline characteristics of this cohort have been published elsewhere [24]. In brief, patients were included if they had typical clinical symptoms of a SARS-CoV-2 infection (e.g., respiratory symptoms with or without fever and/or pulmonary infiltrates) and a positive real-time reverse transcription polymerase chain reaction test (RT-PCR) taken from nasopharyngeal swabs or lower respiratory tract specimens, according to the World Health Organization (WHO) guidance [25]. Written general informed consent was obtained from all analyzed patients. The study was approved by the local ethics committee (EKZN, 2020-01306) and performed in conformance with the Declaration of Helsinki ethical guidelines. For the present analysis, only patients with a complete micronutrient status were included, whereas patients receiving either an oral or intravenous substitution of the analyzed micronutrients at the hospital before obtaining the blood samples were excluded. Patients taking micronutrient supplements at home were included, as we aimed to display micronutrient status at the time of hospital admission.

### 2.2. Data Collection

Clinical data were collected by chart abstraction and automatic export from the electronic health records and included socio-demographics, comorbidities, and pre-existing risk factors for a severe COVID-19 course. Comorbidities were classified according to International Statistical Classification of Diseases and Related Health Problems codes (ICD10). For all patients, age-adjusted Charlson Comorbidity Index [26] and Clinical Frailty Scale [27] were calculated. Patient outcomes, including in-hospital mortality, admission to ICU, need for invasive ventilation, and length of hospital stay (LOS), were collected by chart review. To assess nutritional status, we calculated nutritional risk screening (NRS) 2002 score [28] and body mass index (BMI).

### 2.3. Laboratory Analysis

Laboratory values correspond to blood samples obtained within the first four days of hospitalization. Serum or plasma levels for vitamins A, B12, D, and E, as well as folic acid, zinc, selenium, and copper, were measured. As a surrogate for vitamin A and for vitamin E we measured total retinol and total α-tocopherol concentrations, respectively, by high performance liquid chromatography (HPLC). The method was modified for small sample volume and based on best practice guideline ([29], modified). Vitamin B12 and folates were measured by chemiluminescence microparticle immunoassays on an Abbott Alinity i system. Total vitamin D (cholecalciferol) concentrations were measured by chemiluminescence immunoassay on a DiaSorin LIAISON XL system. Trace elements (copper, selenium, zinc) were measured by inductively coupled plasma mass spectrometry in collision mode (helium). The method was set up without digestion and simple dilution with an alkaline solution containing an internal standard element (rhodium). Table S1 shows cut-off values for deficiencies.

### 2.4. Outcomes

The primary outcome included the assessment of different micronutrient levels and the prevalence of deficiencies in patients hospitalized with COVID-19. Secondary outcomes included the association of micronutrient levels and a composite adverse outcome, defined as ICU admission and/or all-cause in-hospital mortality.

### 2.5. Statistical Analyses

Discrete variables are expressed as frequency (percentage) and continuous variables as medians (interquartile range (IQR)) or means (standard deviation (SD)). Values for vitamin D, vitamin B12, and folic acid were left-censored and values for vitamin B12 and folic acid were right-censored. For statistical analyses, we replaced these values with the corresponding limit values. To test for normal distribution of the analyzed variables, the Shapiro–Wilk test was used. The correlation of different micronutrients was investigated by using a Spearman’s rank correlation analysis and reported as Spearman’s rank coefficient rho with the corresponding *p*-value. Further, we investigated the association of initial micronutrient levels with the composite endpoint of transfer to the ICU and/or all-cause in-hospital mortality with a logistic regression analysis. Odds ratios (OR) including the corresponding 95% confidence intervals (CI) were reported as a measure of association for both micronutrients as continuous and binary (deficient vs. non-deficient) variables. We adjusted the analyses only for age, since a multivariable regression was not possible due to the small sample size, to avoid over-fitting of the model. A two-sided *p*-value of < 0.05 was considered significant. Statistical analyses were performed using Stata, version 15.1 (StataCorp LLC, College Station, TX, USA).

## 3. Results

**Figure 1:**
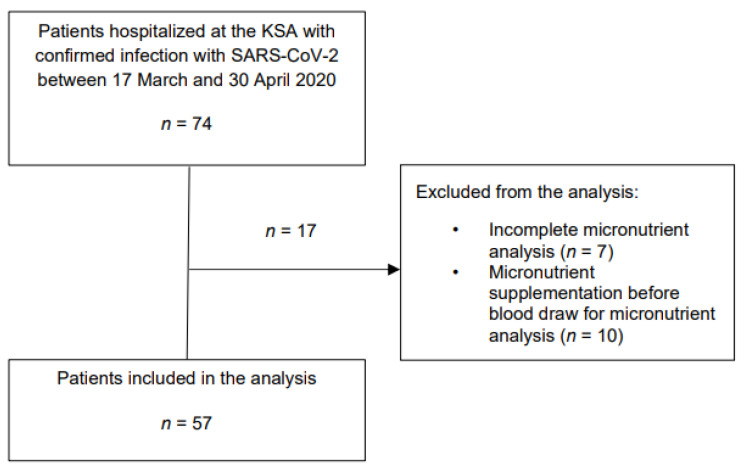
Overview of the study flow.

Overall, 74 patients with confirmed COVID-19 were eligible. Ten patients had to be excluded because of micronutrient substitution before blood draw and seven patients because of incomplete micronutrient values. Therefore, 57 patients were included for the final analysis. Figure 1 provides an overview of the study flow.

### 3.1. Baseline Characteristics

Table 1 shows patient demographics and comorbidities, as well as micronutrient levels and deficiencies, outcomes, and nutritional status stratified by the number of micronutrient deficiencies. Patients were divided into groups with “no”, “one”, “two”, or “multiple” micronutrient deficiencies. In total, a third (*n* = 19, 33%) had three or more (multiple) micronutrient deficiencies and 79% (*n* = 45) at least one deficiency. Among all patients, selenium, vitamin D, vitamin A, and zinc deficiencies were most prevalent (51%, 40%, 39%, and 39%, respectively). Selenium, vitamin D, vitamin A, and zinc levels were lower when patients had more micronutrient deficiencies (*p* < 0.01). In patients with a single micronutrient deficiency, selenium deficiency was most prevalent (*n* = 5, 50%). No patient had a vitamin E deficiency and vitamin B12, folic acid, and copper deficiencies were rare with 7%, 5%, and 2%, respectively.

Median age was 67.0 years (IQR 60.0, 74.2) and 60% (*n* = 34) were male. Age, gender and most comorbidities were relatively equally distributed within the different categories of micronutrient deficiencies. Regarding clinical outcomes of COVID-19, we found higher risks for longer LOS, ICU admissions, and mechanical ventilation with more deficiencies.

### 3.2. Correlation of Different Micronutrient Values

The correlations of different micronutrient levels are shown in Table 2. Significant correlations for vitamin D and folic acid (r = 0.39) were found, as well as for vitamin D and vitamin A (r = 0.27) and vitamin D and selenium (r = 0.32). Further, there was a significant positive correlation for folic acid and selenium (r = 0.3) and vitamin A and zinc (r = 0.42), as well as copper and zinc and copper and selenium (r = 0.37 and 0.29, respectively). All these correlations were moderate, with correlation coefficients in the range of 0.28 to 0.42. A negative correlation was observed between vitamin A and B12 (r = −0.28). No significant correlations were found between vitamin E and other micronutrients.

### 3.3. Association of Micronutrient Levels with ICU Admission and In-Hospital Mortality

In Table 3, micronutrient levels in association with the composite outcome of ICU admission and/or in-hospital mortality in patients with SARS-CoV-2 infection are shown. Median admission folic acid values were over 1.5-fold higher in patients with a mild course of COVID-19 compared to those with a severe course (16.6 nmol/L (IQR 11.4, 24.0) vs. 10.2 nmol/L (IQR 8.2, 14.4), *p* < 0.01). Median vitamin A levels were over two times higher in patients with mild compared to severe courses of COVID-19 (1.5 μmol/L (IQR 1.0, 2.0) vs. 0.7 μmol/L (IQR 0.4, 1.1), *p* < 0.01). Vitamin A and zinc deficiencies were almost threefold more prevalent in patients with severe COVID-19 (73% vs. 26%, *p* < 0.01, 67% vs. 29%, *p* < 0.01, respectively).

**Figure 2:**
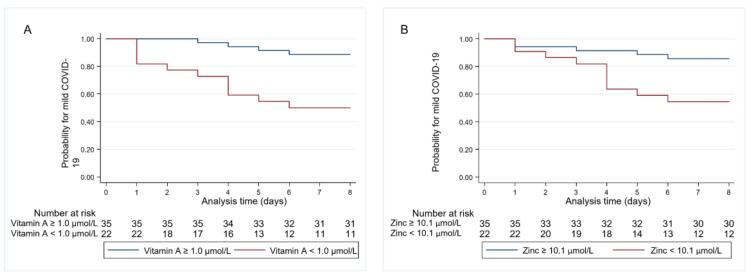
Kaplan–Meier estimates for time to composite endpoint stratified by (**A**) vitamin A deficiency and (**B**) zinc deficiency.

Higher levels of folic acid, vitamin A, or zinc were associated with a lower risk for a severe course of COVID-19 (adjusted OR 0.88 (95% CI 0.78–0.98, *p* = 0.02), adjusted OR 0.18 (95% CI 0.05–0.69, *p* = 0.01), adjusted OR 0.73 (95% CI 0.55–0.98, *p* = 0.03), respectively). Accordingly, both vitamin A and zinc deficiencies were associated with a more than sevenfold higher risk for the composite endpoint of ICU admission and/or in-hospital mortality (adjusted OR 7.41 (95% CI 1.91–29.68, *p* = 0.004, adjusted OR 7.18 (95% CI 1.73–29.76, *p* = 0.007, respectively) (Figure 2).

## 4. Discussion

In this prospective cohort of mostly older patients hospitalized with COVID-19, micronutrient deficiencies were highly prevalent, mainly for selenium, vitamin D, vitamin A, and zinc. There was a high proportion of patients with multiple deficiencies and there were correlations among the different micronutrient levels. Importantly, our results indicate that micronutrient deficiencies are associated with more severe clinical courses of COVID-19 and worse outcomes, especially low levels of folic acid, vitamin A, and zinc.

In our study population, a high prevalence of vitamin D deficiency was found with 40%. This may be partly explained by the seasonal period in which the patients were analyzed (March and April). Data from the Swiss Federal Office of Public Health on vitamin D status show that during the winter period, more than 60% of the population have insufficient vitamin D levels with values < 50 nmol/L [22]. Yet, unlike other studies, we found no difference between vitamin D levels in patients with mild or severe COVID-19 [30,31,32]. Both patients with mild and severe courses of the disease had levels that were only slightly above the cut-off for deficiency (i.e., 30 nmol/L), similar to insufficient vitamin D levels in both patients with COVID-19 and healthy controls in a study by Elham et al. [33]. Compared to our study, studies that found an association of vitamin D deficiency and adverse outcomes in COVID-19 had different outcomes defined, including the need for non-invasive ventilation in one study [15] and a composite of invasive mechanical ventilation and/or death in another study [34]. The lack of an association in our study may be explained by the small sample size with risk for a type II error and the lack of a healthier control group with higher vitamin D baseline levels. Currently, there are several vitamin D treatment trials in COVID-19 ongoing, and it will be interesting to learn whether treatment improves clinical outcomes [35].

Further, our study found a high prevalence of selenium deficiency—in 51% of cases—which is in agreement with data from South Korea, where 42% of COVID-19 patients were selenium deficient [36]. In our study, no association between low selenium levels and adverse clinical outcomes was found. These findings are not consistent with data from China [37] and Belgium [19], but are in agreement with a study from Iran, where an association of selenium deficiency and COVID-19 severity disappeared after adjusting for confounders [20]. Again, the small sample size in our study did not allow us to draw strong conclusions.

Tomasa-Irriguible et al. showed an association of low vitamin A and zinc levels with the need for ICU treatment, but not with mortality in patients with COVID-19 [18]. In a study by Tepasse et al. [38], lower vitamin A levels were associated with acute respiratory distress syndrome and mortality in patients with COVID-19. In our study, the prevalence of vitamin A deficiency was high and vitamin A levels were significantly lower in patients with a severe course of COVID-19 compared to patients with mild courses. Stephensen et al. [10] described that vitamin A levels decrease in the state of inflammation, which could be a reason for low vitamin A levels in COVID-19. However, we found decreased levels only in patients with severe COVID-19. Sarohan et al. [39] hypothesized a possible association of retinoid signaling with the COVID-19 pathogenesis, which may explain the fact that in COVID-19, many different organ systems are affected in a similar way to patients with defects in retinoid signaling. It is well known that vitamin A, through its metabolite retinoid acid, is a regulator in balancing anti- and pro-inflammatory processes, for example regulatory T-cells vs. T-helper-17-cells [40]. There is evidence that this balance is disturbed in severe COVID-19 [41,42], which, therefore, could be partially explained by low vitamin A levels. Further, a study from Bangladesh [43] showed higher interleukin 6 (IL-6) levels in men with low vitamin A stores. Given that high IL-6 levels were observed in patients with severe COVID-19 [44,45], IL-6 receptors are a target in COVID-19 treatment. Two meta-analyses showed that tocilizumab treatment was associated with lower mortality [46,47]. 

Zinc deficiency showed an over sevenfold higher risk for an adverse outcome in COVID-19 in our study. In a French study, low zinc levels were an independent risk factor for hospitalization due to respiratory deterioration in COVID-19 [48] and Du Laing et al. [19] found significantly lower zinc levels in deceased COVID-19 patients in comparison with survivors. However, studies showed that during inflammation, zinc redistribution into hepatocytes is upregulated by cytokines like IL-6, resulting in lower plasma levels [49,50]. Therefore, low zinc levels are possibly a consequence of the infection and may not reflect a pre-existing zinc deficiency. Folic acid levels were almost 1.5 times higher in mild versus severe courses of COVID-19. However, levels in patients with a clinical progression to severe COVID-19 were only slightly below the cut-off values, so the clinical relevance may be marginal.

In our study, we found a stepwise increase in LOS and the need for ICU care in patients with a higher number of deficiencies. However, the causality of these observations is unclear. It is known that infections may lead to micronutrient deficiencies via various mechanisms, for example lower intake, malabsorption, and redistribution in the body [51]. Therefore, it remains unclear, if deficiencies in micronutrients are a risk factor for infections and a more severe disease course, or if the infection itself leads to the deficiencies due to higher need.

In addition, the question of whether deficiencies occur in a specific pattern was analyzed. Indeed, we found multiple positive correlations among different micronutrient levels with moderate correlation coefficients between r = 0.27 and 0.42, suggesting that the risk for additional deficiencies is increased if one such deficiency is found. This finding is in line with a study from China that included pediatric patients with respiratory infections [52] and showed correlations among vitamin A, vitamin D, and vitamin E. As a consequence, it may be advised to supplement different micronutrients at the same time using a multivitamin drug instead of only focusing on single micronutrients. 

There are several limitations for this study. Firstly, it was a single-center study with a relatively small number of patients. Due to the small sample size, adjusting for multiple confounders was not possible. Secondly, the analyzed blood samples were not obtained on the day of admission but during the first four days of hospitalization. This may have led to slight differences compared to admission levels. Therefore, we excluded patients who already received micronutrient supplementation before the blood draw. In two patients, the beginning of enteral nutrition by stomach tube was not clearly documented. Given that we did not consider food intake as a supplementation and recent data showed no association between standard enteral nutrition and micronutrients levels in patients in the ICU [53], we decided to include these two patients in the analysis. A further limitation is that blood collection was independent of fasting state. Additionally, some patients received micronutrient supplements after the blood draw, especially if hospitalized in ICU, which made the interpretation of data more difficult. Lastly, our study did not include a healthy control group in order to better understand the significance of micronutrient deficiency in patients with COVID-19.

In conclusion, micronutrient deficiencies are common in patients with COVID-19. This study is in line with previous data and shows an association of micronutrient deficiencies and adverse outcomes in COVID-19. As the link between a well-functioning immune system and sufficient levels of micronutrients is well described, further research is warranted to assess the benefits of micronutrient supplementation for either prevention or treatment of COVID-19. 

## Figures and Tables

**Table 1:** Baseline characteristics and micronutrient levels stratified by number of micronutrient deficiencies.

	All	No Deficiency	One Deficiency	Two Deficiencies	Multiple Deficiencies	*p*-Value °
	*n* = 57	*n* = 12	*n* = 10	*n* = 16	*n* = 19	
Socio-demographics						
Age (years), median (IQR)	67.0 (60.0, 74.2)	64.5 (57.8, 74.3)	71.0 (65.1, 72.4)	63.6 (58.9, 73.4)	67.5 (55.9, 76.6)	0.87
Gender (male), *n* (%)	34 (60)	6 (50)	7 (70)	10 (63)	11 (58)	0.80
Nationality						
Swiss, *n* (%)	37 (65)	9 (75)	8 (80)	11 (69)	9 (47)	0.46
Others, *n* (%)	13 (23)	3 (25)	1 (10)	3 (19)	6 (32)	
Unknown, *n* (%)	7 (12)	0 (0)	1 (10)	2 (13)	4 (21)	
Pre-existing risk-factors						
Active smoker, *n* (%)	5 (12)	1 (11)	1 (14)	1 (7)	2 (15)	0.92
Immunosuppressant, *n* (%)	1 (2)	0 (0)	1 (10)	0 (0)	0 (0)	0.19
Pre-admission history						
Transfer from another hospital, *n* (%)	14 (25)	0 (0)	1 (10)	6 (38)	7 (37)	0.05
Symptom onset before admission (days), median (IQR)	7.0 (5.0, 11.0)	8.0 (6.0, 11.0)	9.0 (6.0, 14.0)	7.5 (4.5, 12.5)	5.5 (3.0, 9.0)	0.38
Comorbidities						
Cancer, *n* (%)	5 (9)	0 (0)	1 (10)	2 (13)	2 (11)	0.68
Hypertension, *n* (%)	35 (61)	6 (50)	7 (70)	11 (69)	11 (58)	0.70
Coronary artery disease, *n* (%)	16 (28)	3 (25)	6 (60)	3 (19)	4 (21)	0.10
Chronic heart failure, *n* (%)	3 (5)	1 (8)	2 (20)	0 (0)	0 (0)	0.09
Asthma, *n* (%)	11 (19)	2 (17)	3 (30)	3 (19)	3 (16)	0.82
Chronic obstructive pulmonary disease, *n* (%)	3 (5)	0 (0)	2 (20)	0 (0)	1 (5)	0.12
Obstructive sleep apnea syndrome, *n* (%)	10 (18)	2 (17)	3 (30)	2 (13)	3 (16)	0.71
Active rheumatic disease, *n* (%)	1 (2)	0 (0)	1 (10)	0 (0)	0 (0)	0.19
Chronic kidney disease, *n* (%)	17 (30)	3 (25)	5 (50)	5 (31)	4 (21)	0.42
Diabetes, *n* (%)	18 (32)	4 (33)	5 (50)	3 (19)	6 (32)	0.42
Age-adjusted Charlson comorbidity index, median (IQR)	3.0 (2.0, 6.0)	2.0 (1.0, 6.5)	6.5 (2.0, 9.0)	3.0 (2.0, 5.0)	3.0 (2.0, 5.0)	0.21
Clinical frailty score, median (IQR)	3.0 (2.0, 4.0)	2.5 (2.0, 3.5)	3.0 (3.0, 5.0)	3.0 (3.0, 5.0)	3.0 (2.0, 4.0)	0.51
Outcomes						
Length of hospital stay (days), median (IQR)	9.0 (5.0, 14.0)	5.0 (4.0, 8.5)	5.0 (4.0, 10.0)	10.0 (8.0, 11.5)	19.0 (6.0, 22.0)	0.02
ICU admission, *n* (%)	12 (21)	0 (0)	0 (0)	3 (19)	9 (47)	<0.01
Need for mechanical ventilation, *n* (%)	10 (18)	0 (0)	0 (0)	2 (13)	8 (42)	<0.01
In-hospital death, *n* (%)	6 (11)	0 (0)	3 (30)	1 (6)	2 (11)	0.12
Micronutrients						
Vitamin A						
Median (μmol/L), (IQR)	1.2 (0.8, 1.7)	1.8 (1.6, 2.5)	1.9 (1.4, 2.4)	1.0 (0.7, 1.4)	0.8 (0.6, 1.1)	<0.01
Deficiency, *n* (%)	22 (39)	0 (0)	1 (10)	8 (50)	13 (68)	<0.01
Vitamin B12						
Median (pmol/L), (IQR)	292.0 (207.0, 548.0)	255.0 (213.0, 560.0)	268.0 (171.0, 493.0)	545.0 (335.5, 706.0)	237.0 (182.0, 310.0)	0.04
Deficiency, *n* (%)	4 (7)	0 (0)	0 (0)	1 (6)	3 (16)	0.27
Vitamin D						
Median (nmol/L), (IQR)	34.6 (24.3, 62.2)	61.0 (51.2, 80.2)	65.0 (36.4, 70.0)	32.4 (26.8, 52.0)	23.6 (15.2, 31.5)	<0.01
Deficiency, *n* (%)	23 (40)	0 (0)	2 (20)	7 (44)	14 (74)	<0.01
Vitamin E						
Mean (μmol/L), (SD)	32.9 (8.6)	35.7 (7.5)	31.6 (8.3)	35.2 (10.1)	30.0 (7.7)	0.19
Deficiency, *n* (%)	0 (0)	0 (0)	0 (0)	0 (0)	0 (0)	n.a.
Folic acid						
Median (nmol/L), (IQR)	15.0 (10.2, 23.3)	18.4 (13.6, 23.9)	19.6 (11.7, 27.6)	15.3 (9.7, 21.6)	11.8 (8.2, 17.9)	0.07
Deficiency, *n* (%)	3 (5)	0 (0)	0 (0)	0 (0)	3 (16)	0.10
Zinc						
Median (μmol/L), (IQR)	10.9 (8.8, 12.8)	13.9 (12.4, 16.0)	12.6 (11.4, 14.4)	10.7 (8.5, 11.7)	9.3 (8.3, 10.7)	<0.01
Deficiency, *n* (%)	22 (39)	0 (0)	2 (20)	7 (44)	13 (68)	<0.01
Selenium						
Mean (μmol/L), (SD)	0.96 (0.29)	1.20 (0.18)	1.06 (0.28)	0.92 (0.26)	0.78 (0.24)	<0.01
Deficiency, *n* (%)	29 (51)	0 (0)	5 (50)	9 (56)	15 (79)	<0.01
Copper						
Mean (μmol/L), (SD)	21.2 (4.0)	21.3 (3.3)	22.1 (3.6)	21.3 (4.4)	20.7 (4.4)	0.85
Deficiency, *n* (%)	1 (2)	0 (0)	0 (0)	0 (0)	1 (5)	0.57
Nutritional assessment						
NRS ≥ 3, *n* (%)	8 (19)	0 (0)	3 (38)	2 (20)	3 (19)	0.27
BMI						
18.5–24.9 kg/m^2^, *n* (%)	15 (33)	3 (33)	0 (0)	6 (46)	6 (38)	0.36
25–29.9 kg/m^2^, *n* (%)	17 (37)	4 (44)	5 (63)	4 (31)	4 (25)	
≥30 kg/m^2^, *n* (%)	14 (30)	2 (22)	3 (38)	3 (23)	6 (38)	

° ANOVA for normally distributed variables, Kruskal–Wallis for continuous variables, Pearson’s chi squared test for binary and categorical variables. Abbreviations: BMI, body mass index; IQR, interquartile range; NRS, nutritional risk score; SD, standard deviation.

**Table 2:** Spearman’s rank correlation among micronutrient levels.

	Vitamin A	Vitamin B12	Vitamin D	Vitamin E	Folic Acid	Zinc	Selenium	Copper
Vitamin A	1							
Vitamin B12	**−0.28, *p* = 0.04**	1						
Vitamin D	**0.27, *p* = 0.04**	−0.04, *p* = 0.75	1					
Vitamin E	0.24, *p* = 0.08	0.19, *p* = 0.15	0.08, *p* = 0.56	1				
Folic acid	0.21, *p* = 0.11	−0.008, *p* = 0.95	**0.39, *p* = 0.002**	0.04, *p* = 0.79	1			
Zinc	**0.42, *p* = 0.001**	−0,17, *p* = 0.22	0.19, *p* = 0.16	0.18, *p* = 0.18	−0.03, *p* = 0.83	1		
Selenium	0.20, *p* = 0.13	0.07, *p* = 0.59	**0.32, *p* = 0.02**	0.25, *p* = 0.06	**0.30, *p* = 0.02**	0.26, *p* = 0.05	1	
Copper	−0.07, *p* = 0.58	−0.04, *p* = 0.75	−0.14, *p* = 0.28	0.26, *p* = 0.05	0.05, *p* = 0.72	**0.37, *p* = 0.004**	**0.29, *p* = 0.03**	1

Bold if statistically significant.

**Table 3:** Micronutrient levels stratified by composite endpoint and crude and adjusted association of micronutrient levels and composite endpoint of ICU admission and/or in-hospital mortality.

	Mild Disease	Severe Disease	*p*-Value °	Univariable OR (95% CI), *p*-Value	Adjusted OR * (95% CI), *p*-Value
	*n* = 42	*n* = 15			
*Vitamin A*					
Median (μmol/L), (IQR)	1.5 (1.0, 2.0)	0.7 (0.4, 1.1)	<0.01	0.17 (0.05–0.66), ***p* = 0.01**	0.18 (0.05–0.69), ***p* = 0.01**
Deficiency, *n* (%)	11 (26)	11 (73)	<0.01	7.75 (2.04–29.46), ***p* = 0.003**	7.41 (1.91–28.68), ***p* = 0.004**
*Vitamin B12*					
Median (pmol/L), (IQR)	290.0 (200.0, 597.0)	310.0 (220.0, 497.0)	0.82	1.00 (0.99–1.00), *p* = 0.91	1.00 (0.99–1.00), *p* = 0.91
Deficiency, *n* (%)	4 (10)	0 (0)	0.22	n.a.	n.a.
*Vitamin D*					
Median (nmol/L), (IQR)	34.4 (24.3, 65.0)	34.6 (16.2, 46.8)	0.31	0.99 (0.96–1.01), *p* = 0.32	0.99 (0.96–1.01), *p* = 0.38
Deficiency, *n* (%)	16 (38)	7 (47)	0.56	1.42 (0.43–4.68), *p* = 0.56	1.44 (0.43–4.79), *p* = 0.55
*Vitamin E*					
Mean (μmol/L), (SD)	33.1 (9.0)	32.5 (7.7)	0.81	0.99 (0.92–1.06), *p* = 0.81	0.99 (0.93–1.07), *p* = 0.88
Deficiency, *n* (%)	0 (0)	0 (0)	n.a.	n.a.	n.a.
*Folic acid*					
Median (nmol/L), (IQR)	16.6 (11.4, 24.0)	10.2 (8.2, 14.4)	<0.01	0.88 (0.79–0.98), ***p* = 0.02**	0.88 (0.78–0.98), ***p* = 0.02**
Deficiency, *n* (%)	0 (0)	3 (20)	<0.01	n.a.	n.a.
*Zinc*					
Median (μmol/L), (IQR)	11.7 (9.8, 13.5)	9.3 (8.3, 11.4)	0.03	0.77 (0.60–0.99), ***p* = 0.04**	0.73 (0.55–0.98), ***p* = 0.03**
Deficiency, *n* (%)	12 (29)	10 (67)	<0.01	5 (1.41–17.72), ***p* = 0.01**	7.18 (1.73–29.76), ***p* = 0.007**
*Selenium*					
Mean (μmol/L), (SD)	0.9 (0.3)	1.0 (0.3)	0.66	1.62 (0.20–13.11), *p* = 0.65	1.39 (0.16–12.27), *p* = 0.77
Deficiency, *n* (%)	21 (50)	8 (53)	0.82	1.14 (0.35–3.72), *p* = 0.83	1.19 (0.36–3.93), *p* = 0.77
*Copper*					
Mean (μmol/L), (SD)	21.2 (4.4)	21.3 (2.9)	0.93	1.00 (0.87–1.17), *p* = 0.92	1.00 (0.86–1.16), *p* = 0.99
Deficiency, *n* (%)	1 (2)	0 (0)	0.55	n.a.	n.a.

° ANOVA for normally distributed variables, Wilcoxon rank-sum test for continuous variables, * adjusted for age. Bold if statistically significant. Abbreviations: CI, confidence interval; ICU, intensive care unit; IQR, interquartile range; SD, standard deviation; n.a., not applicable; OR, odds ratio.

## Data Availability

The data presented in this study are available on request from the corresponding author.

## Supplementary Materials

The following are available online at https://www.mdpi.com/article/10.3390/nu14091862/s1, Table S1: Cut-off values for different micronutrients [54].
