# Supplementary material for: Prevalence of Micronutrient Deficiencies in Patients Hospitalized with COVID-19: An Observational Cohort Study
Source: Nutrients. 2022 Apr 29;14(9):1862. doi: 10.3390/nu14091862 (PMC9101904; doi:10.3390/nu14091862)
Supplement: Supplementary file 1 [file nutrients-14-01862-s001.zip › nutrients-1682608-supplementary.pdf]

Table S1. Cut-off values for different micronutrients

Parameter	Cut-off
Vitamin A	< 1.0 µmol/l
Vitamin B12	< 140 pmol/l
Vitamin D	< 30.0 nmol/l
Vitamin E	< 11.6 µmol/l
Folic acid	< 7.0 nmol/l
Zinc	< 10.1 µmol/l
Selenium	< 0.94 µmol/l
Copper	< 11.8 µmol/l

Cut-off values were based on the reference range of the laboratory at the Cantonal Hospital Aarau, whereas the cut-off value for vitamin D was according to Munns [1].

1. Munns, C.F.; Shaw, N.; Kiely, M.; Specker, B.L.; Thacher, T.D.; Ozono, K.; Michigami, T.; Tiosano, D.; Mughal, M.Z.; Mäkitie, O.; et al. Global Consensus Recommendations on Prevention and Management of Nutritional Rickets. *J. Clin. Endocrinol. Metab.* **2016**, *101*, 394–415.
